# Supplementary material for: Weighted Gene Co-Expression Network Analysis Uncovers Core Drought Responsive Genes in Pecan (Carya illinoinensis)
Source: Plants (Basel). 2025 Mar 7;14(6):833. doi: 10.3390/plants14060833 (PMC11944766; doi:10.3390/plants14060833)
Supplement: Supplementary file 1 [file plants-14-00833-s001.zip › Figure S1.pdf]

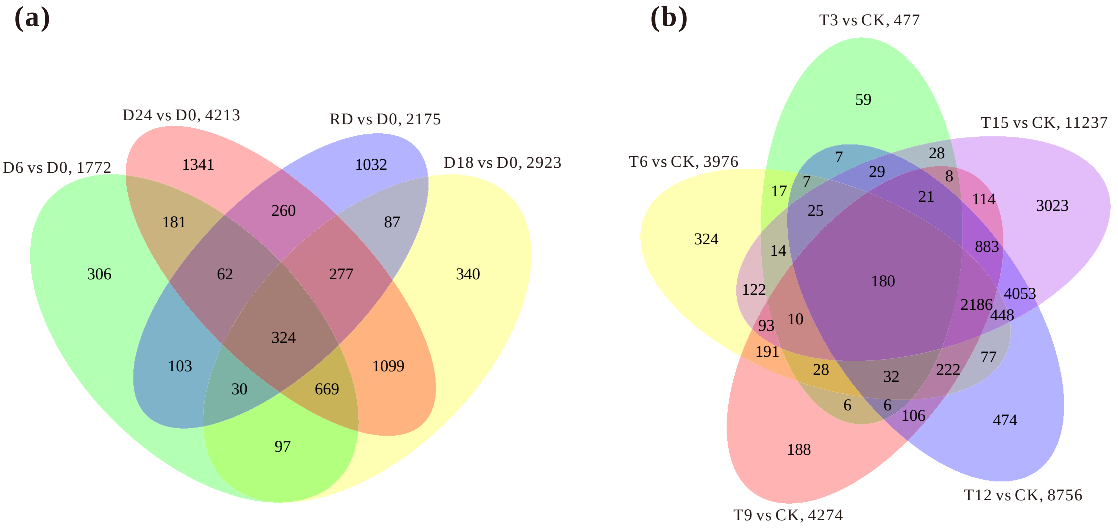

Figure S1. The number differentially expressed genes (DEGs) for pecan in response to drought stress derived from two different projects (P1 and P2). (A) The number of DEGs for P1. D0~D24 represent the samples collected 0~24 days after drought stress. RD is the sample collected after rehydration. (B) The number of DEGs for P2. CK~T15 represent the samples collected 0~15 days after drought stress
