# Supplementary material for: Weighted Gene Co-Expression Network Analysis Uncovers Core Drought Responsive Genes in Pecan (Carya illinoinensis)
Source: Plants (Basel). 2025 Mar 7;14(6):833. doi: 10.3390/plants14060833 (PMC11944766; doi:10.3390/plants14060833)
Supplement: Supplementary file 1 [file plants-14-00833-s001.zip › Figure S2.pdf]

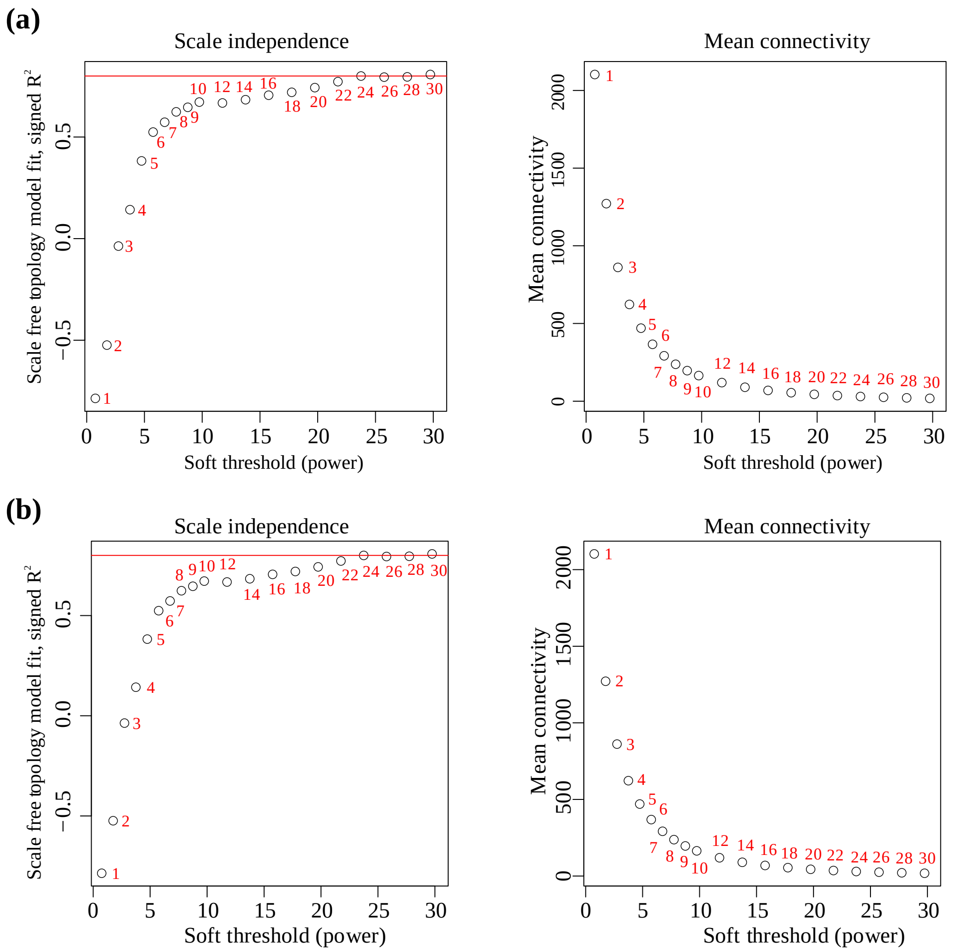

Figure S2. Proper soft threshold detection for the two different projects (P1 and P2). (**a**) Analysis of the scale-free topology model fit index (signed R^2) and mean connectivity for multiple soft threshold values in P1. (**b**) Analysis of signed R^2 and mean connectivity for multiple soft threshold values in P2.
